# Analysis of Gun-Cotton

**Published:** 1847-04

**Authors:** 


*Analysis of Gun-Cotton.*—In a late English paper I observed an analysis of gun-cotton made by Professor Graham, of London. Exploded in a glass tube so as to collect the gaseous products, 53.33 grains of the cotton yielded 100 cubic inches of gas, of which the composition was as follows:

| | |
|---|---|
| Carbonic acid, . . . . | 14.284, or 2 volumes; |
| Cyanogen, . . . . | 7.143, or 1 volume ; |
| Nitric oxide, . . . . | 35.715, or 5 volumes; |
| Carbonic oxide, . . . . | 35.715, or 5 volumes; |
| Nitrogen . . . . | 7.134, or 1 volume. |

Besides these aëriforn bodies, a portion of oxalic acid is precipitated, and a considerable quantity of water results from the combustion.
*Ibid.*

